# Genome mining reveals polysaccharide-degrading potential and new antimicrobial gene clusters of novel intestinal bacterium *Paenibacillus jilinensis* sp. nov.

**DOI:** 10.1186/s12864-022-08623-4

**Published:** 2022-05-19

**Authors:** Ke Ma, Wei Chen, Shi-Qing Yan, Xiao-Qi Lin, Zhen-Zhen Liu, Jia-Bao Zhang, Yu Gao, Yong-Jun Yang

**Affiliations:** Key Laboratory of Zoonosis Research, Ministry of Education, College of Veterinary Medicine, Jilin University, No. 5333 Xi’an Road, Changchun, 130062 China

**Keywords:** *Paenibacillus jilinensis* sp. nov., Genome analysis, CAZymes, BGCs, Antibacterial activity

## Abstract

**Background:**

Drug-resistant bacteria have posed a great threat to animal breeding and human health. It is obviously urgent to develop new antibiotics that can effectively combat drug-resistant bacteria. The commensal flora inhabited in the intestines become potential candidates owing to the production of a wide range of antimicrobial substances. In addition, host genomes do not encode most of the enzymes needed to degrade dietary structural polysaccharides. The decomposition of these polysaccharides mainly depends on gut commensal-derived CAZymes.

**Results:**

We report a novel species isolated from the chicken intestine, designated as *Paenibacillus jilinensis* sp. nov. and with YPG26^T^ (= CCTCC M2020899^T^) as the type strain. The complete genome of *P. jilinensis* YPG26^T^ is made up of a single circular chromosome measuring 3.97 Mb in length and containing 49.34% (mol%) G + C. It carries 33 rRNA genes, 89 tRNA genes, and 3871 protein-coding genes, among which abundant carbohydrate-degrading enzymes (CAZymes) are encoded. Moreover, this strain has the capability to antagonize multiple pathogens in vitro. We identified putative 6 BGCs encoding bacteriocin, NRPs, PKs, terpenes, and protcusin by genome mining. In addition, antibiotic susceptibility testing showed sensitivity to all antibiotics tested.

**Conclusions:**

This study highlights the varieties of CAZymes genes and BGCs in the genome of *Paenibacillus jilinensis*. These findings confirm the beneficial function of the gut microbiota and also provide a promising candidate for the development of new carbohydrate degrading enzymes and antibacterial agents.

**Supplementary Information:**

The online version contains supplementary material available at 10.1186/s12864-022-08623-4.

## Background

The gut microbiota plays a crucial role in the host physiology, metabolism, immunity, digestion, and nutrition uptake, but most of these microorganisms are still uncultivable due to certain limiting factors [1]. Access to these uncultivated species has the potential to significantly advance our understanding of intestinal flora. Delightfully, some recently developed approaches that isolate uncultivated microorganisms, such as situ cultivation [2] and culturomics [3], have proven successful.

Carbohydrates derived from plant polysaccharides affect the stability of the gut microbiota and restructure the composition and metabolism of the gut community, while has a profound effect on human and animal health [4]. As host genomes do not encode most enzymes needed to degrade dietary structural polysaccharides, the decomposition of these polysaccharides mainly depends on gut commensal-derived CAZymes [5]. In addition to degrading structural carbohydrates, gut commensals can also antagonize enteric pathogens by producing a wide range of antimicrobial compounds [6]. Based on their biosynthesis pathway, they are classified into three main groups: bacteriocins, nonribosomal peptides (NRPs), and polyketides (PKs) [7]. These antimicrobial substances are promising candidates for the development of new anti-infective agents.

Here, we report the isolation of an uncultured *Paenibacillus* species, *P. jilinensis* YPG26, isolated from the chicken intestine. We describe its genome characteristics and further identify novel carbohydrate degradation enzyme genes and biosynthetic gene clusters (BGCs) potentially involved in pathogen antagonism.

## Results and discussion

### Isolation, identification, and phylogenetic analysis

**Fig. 1:**
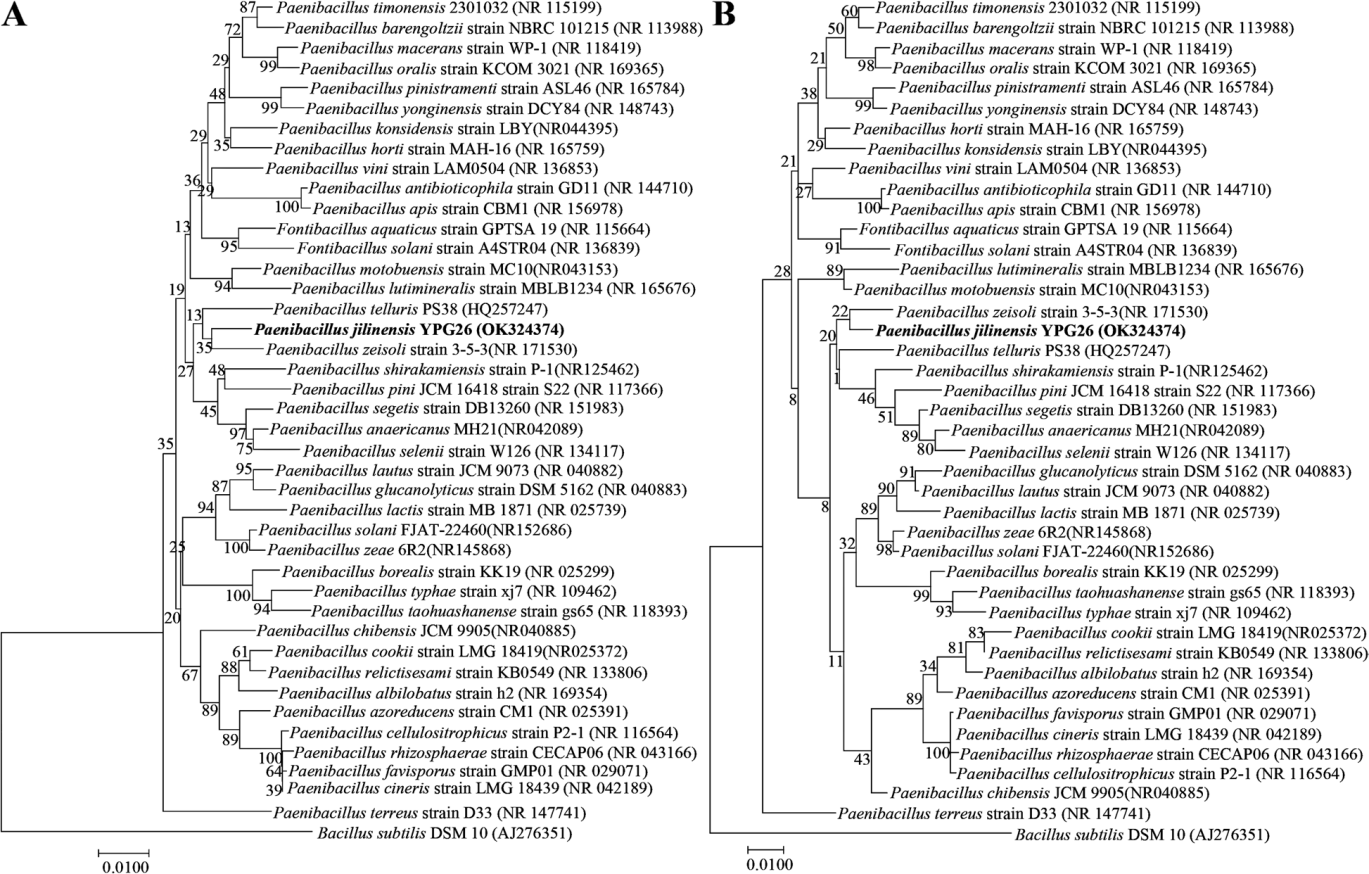
Phylogenetic analysis of the strain YPG26^T^ Neighbor-joining (**A**) and maximum likelihood (**B**) phylogenetic tree based on the 16S rRNA gene sequences of the strain YPG26^T^ (1384 bp) showed the taxonomic position of the strain YPG26^T^ and closely related taxa. Bootstrap values (percentages of 1000 replications) are shown at branch points. *Bacillus subtilis* DSM 10^ T^ (GenBank accession no. AJ276351) was used as outgroup. The bar, 0.01 nucleotide substitutions per site

Bacteria of the genus *Paenibacillus* have been isolated from a variety of environments, such as humans, animals, plants, and soil [8]. The bacterial strain YPG26^T^ was isolated from the chicken intestine. The phylogenetic tree was constructed using the neighbor-joining method and the maximum likelihood method with 1000 bootstrap replications based on the 16S rRNA gene sequence. It showed the strain YPG26^T^ was assigned to the genus *Paenibacillus* and was closest related to the species *Paenibacillus telluris* PS38^T^ (GenBank accession no. HQ257247) with 97.61% similarity, but the strain YPG26^T^ formed a distinct phylogenetic branch within the genus *Paenibacillus* (Fig. 1A). The same relationship was also supported in trees reconstructed using the maximum likelihood method (Fig. 1B). In general, the recommended 16S rRNA sequence similarity thresholds for bacterial genus and species identification were 95% and 98.65%, respectively [9]. Therefore, based on the classification threshold, the strain YPG26^T^ should be assigned as a novel species of *Paenibacillus*. The accession number for the 16S rRNA gene sequence of the strain YPG26^T^ deposited in the GenBank database is OK324374.

### Phenotypic characteristics

*Paenibacillus* Species can be gram-negative, gram-positive, or gram variable, and have different growth status on the same medium (the same as *P. glycanilyticus* CCI5 [10], the strain YPG26^T^ cannot grow on LB medium, while *Paenibacillus alvei* MP1 can grow on LB medium [11]).The strain YPG26^T^ formed a single separated colony on TSB agar plate that was 1–1.5 mm in diameter, circular with slightly irregular edges, grayish-white, low convex, translucent, and glossy after aerobic cultivation at 37℃ for 20 h (Fig. S1A). The colony was slightly different in anaerobic culture (Fig. S1B). By transmission electron microscope observation, bacterial cells were rods approximately 0.9–1.2 μm wide and 4.0–5.0 μm long (Fig. S1C). Gram staining showed that the strain was positive (Fig. S1D). Growth was observed at pH levels ranging from 5–8, with an optimum pH at 8.0, temperatures ranging from 15–50 °C, with an optimum growth at 37 °C, and the strain tolerated NaCl concentration of up to 2.0% (Fig. S1E-G). Other physiological and biochemical characteristics are provided in Table 1, and the attributes of reference species are also described together (Table 1).

**Table 1:** Phenotypic characteristics of the strain YPG26^T^ and type strains of phylogenetically related Paenibacillus species

Phenotypic characteristics	YPG26	1	2	3
isolation from	Chicken intestine	Soil	Soil	Shirakami Mountains
DNA G + C content (mol%)	49.3	49.5	52.8	43.9
Cell size (μm)	0.9–1.2 × 4.0–5.0	0.7–1.0 × 4.0–5.0	0.5–0.8 × 3–5	0.8 × 2–5
Gram staining	+	+	+	-
pH optimum (range)	7.0(5.0–8.0)	7.0 (6.0–9.0)	7.0 (4.5–9.0)	6.5(5.0–8.0)
Temperature (℃) optimum (range)	37(15–50)	37(15–45)	37(10–50)	25(4–35)
Growth at 5% NaCl	-	+	-	-
Anaerobic growth	+	+	-	-
Nitrate reduction	+	+	+	-
ONPG test	-	-	+	+
H_2_S production	-	-	-	-
Utilization of glucose	+	+	-	+
Utilization of maltose	+	+	-	+
Hydrolysis of starch	+	ND	+	-
Catalase	+	+	+	+
Oxidase	+	+	+	+
Arginine dihydrolase	-	ND	ND	ND
Lysine decarboxylase	-	ND	ND	ND
Hydrolysis of urea	+	-	-	-

Type strains 1, *Paenibacillus telluris* PS38^T^; 2, *Paenibacillus chibensis* JCM 9905^ T^; 3, *Paenibacillus shirakamiensis* P-1^ T^. Type strains result were taken from Lee et al. [12], Shida et al. [13], Tonouchi et al. [14], respectively. + positive, - negative, *ND *not detection

### Genomic properties, ANI and DDH analyses

**Fig. 2:**
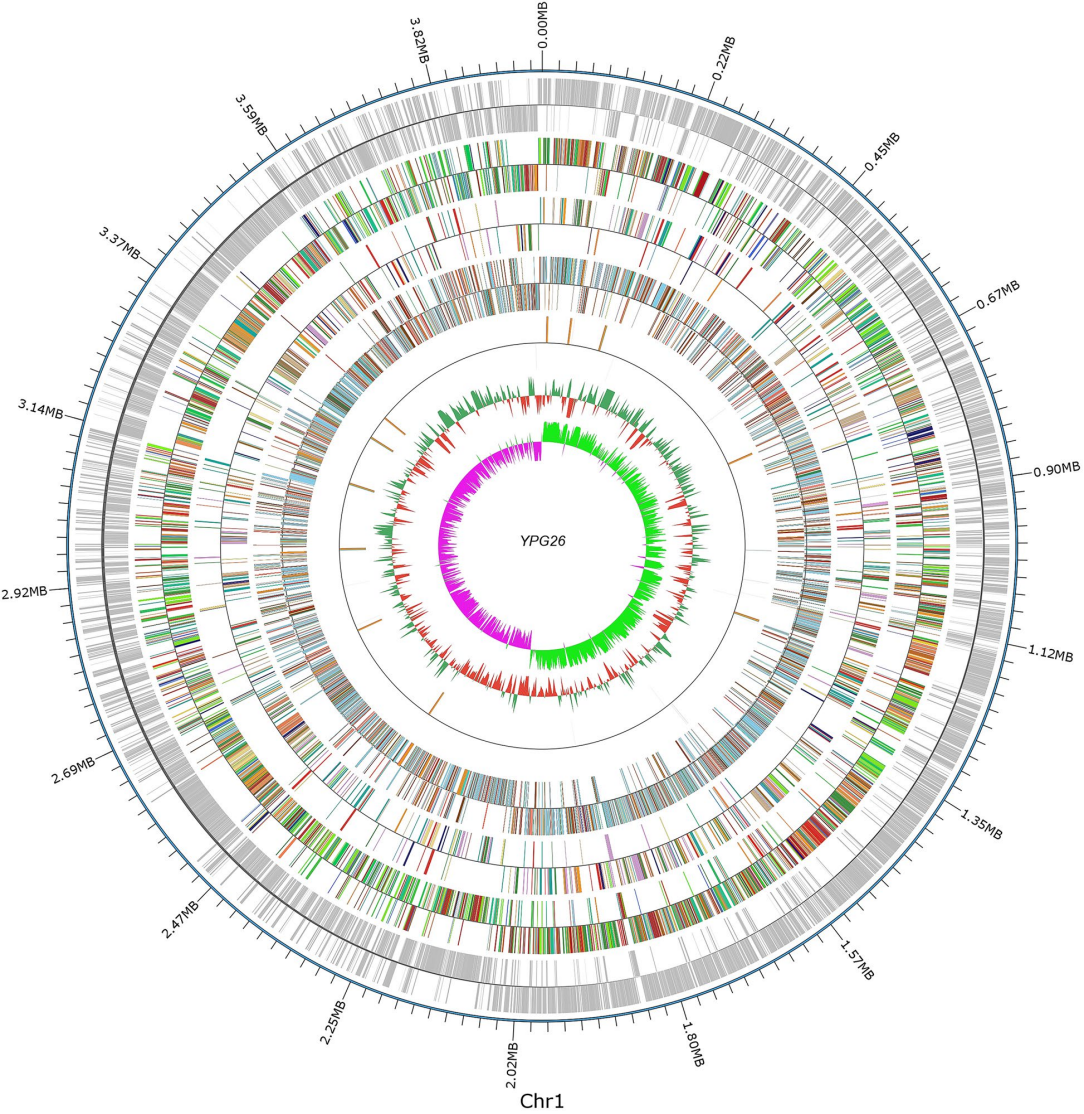
Circular representation of the strain YPG26^T^ genome From the outside to the inside: circle 1, genomic position in kb; circles 2, protein-coding sequences (CDS) on the forward and the reverse strands; circles 3 and 5, COG, GO functional classification of protein-coding genes on forward and reverse strands, respectively; circles 6, non-coding RNA; circles 7, G + C content, green (outward) and red (inward) indicate higher and lower than average value of 49.34%, respectively; circles 8, GC skew ([G—C]/[G + C]), pink (outward) and light green (inward) denote positive (leading strand) and negative (lagging strand) values, respectively

The generated complete genome of the strain YPG26^T^ was composed of a 3,966,665 bp circular chromosome (Fig. 2) with a G + C content of 49.34% (mol%), which fit the range of the genome size of Paenibacillus from 3.02 Mbp (P. darwinianus Br) to 8.82 Mbp (P. mucilaginosus K02) and G + C content from 39 to 59 mol% [8]. It carried 33 rRNA genes, 89 tRNA genes, and 3871 protein-coding genes (CDSs) (Table 2). The functional gene annotation was performed by blasting predicted genes against the COG and GO databases (Fig. S2).

**Table 2:** Genomic characteristics of the strain YPG26^T^

Characteristics	YPG26
Genome size (Mb)	3.97
Protein coding genes	3871
DNA G + C content (mol%)	49.3
Genes assigned to COGs	2628
Genes assigned to GO	2536
rRNA genes	33
tRNA genes	89
Genomics islands number	7
Prophages number	0
CRISPRs number	5
Plasmids number	1

Taxonomic and functional research of microorganisms has increasingly relied upon genome-based data and methods [15]. DNA-DNA hybridization (DDH) and average nucleotide identity (ANI) have become two gold standards for prokaryotic species circumscriptions at the genomic level [16]. The DDH and ANI values between the strain YPG26^T^ and reference species of *Paenibacillus* ranged from 13.2 to 14.0% and 67.56% to 71.07%, respectively (Table S1), which were well below the proposed thresholds of 70% and 95% for prokaryotic species delineation[17, 18]. The results of the genome analysis were consistent with the outcome of the 16S rRNA sequence-based phylogenetic analysis. It also confirmed that the strain YPG26^T^ was a novel *Paenibacillus* species at the genome level, which was suggested to be named *Paenibacillus jilinensis* sp. nov. (jilinensis pertaining to Jilin, a province in northeast China). The type strain is YPG26^T^ (= CCTCC M2020899^T^). The whole-genome sequence of *P. jilinensis* YPG26^T^ has been deposited on the GenBank database with accession number CP084530.

### Identification of carbohydrate-active enzymes

**Fig. 3:**
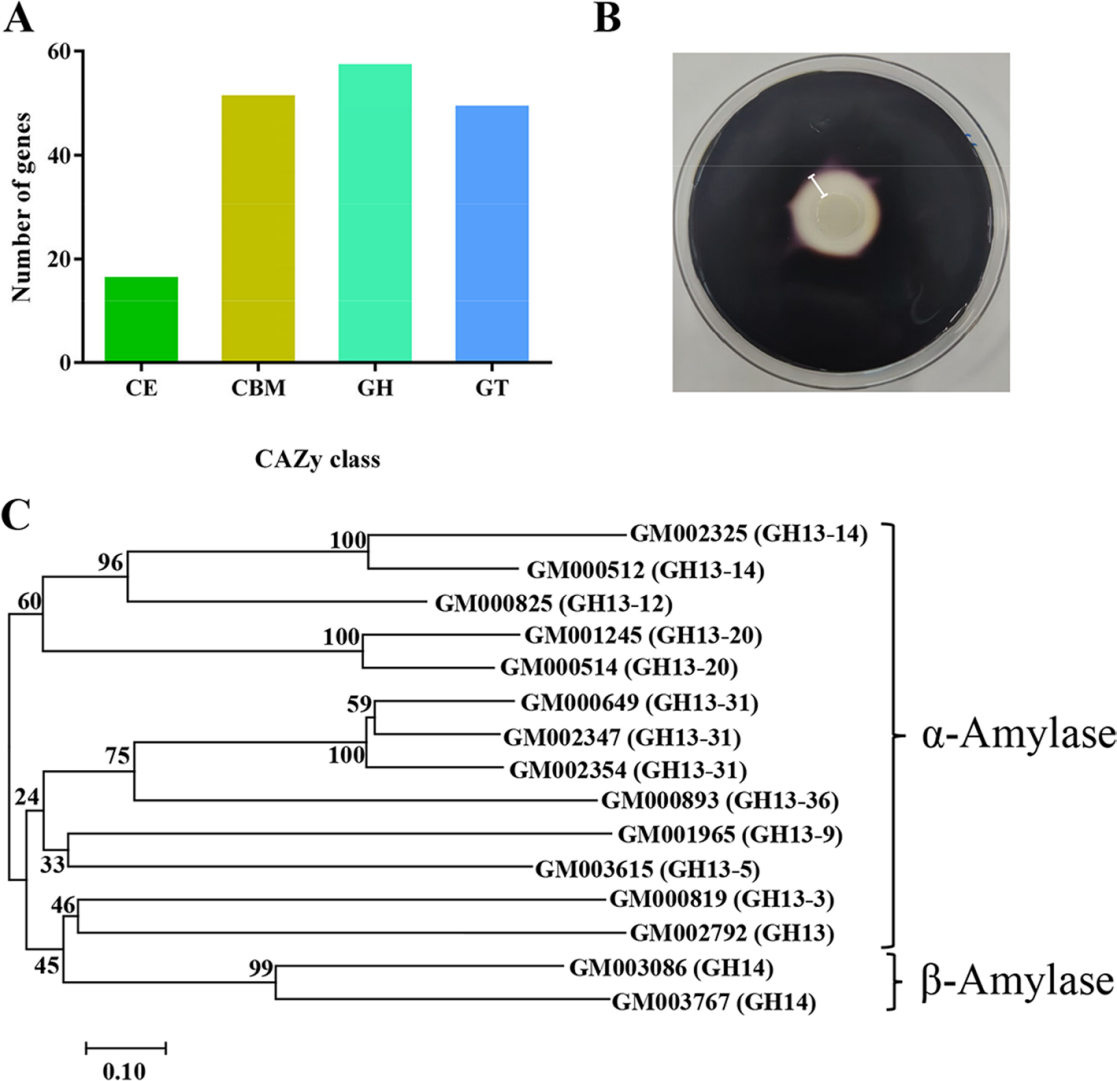
CAZymes distribution and starch degrading enzyme activity of the strain YPG26^T^ (**A**) Number of identified CAZymes families in the genomes of the strain YPG26^T^; **B** Starch degrading enzyme activity on starch agar plate (an area of clearance around a bacterial colony can be observed); **C** Phylogenetic analysis of predicted α- and β-amylases from the strain YPG26^T^

Carbohydrate-active enzymes (CAZymes) are responsible for the biosynthesis, modification, and degradation of carbohydrates and glycoconjugates. They are involved in many metabolic pathways and are essential for microorganisms' survival [19]. By analyzing 41 *Paenibacillus* genomes comprising 25 species, Huang WC et al. found *Paebacillus* genomes encode a wide repertoire of CAZymes [20]. Three CAZymes classes were predicted in the genome of *P. jilinensis* YPG26^T^ using the carbohydrate-active enzymes database (CAZy). Glycoside hydrolases (GHs) were the most abundant class, with 58 predicted domains, followed by 50 glycosyl transferases (GTs) and 17 carbohydrate esterases (CEs) (Fig. 3A). Moreover, a total of 52 putative carbohydrate binding modules (CBMs) were present in the genomic sequence, which are appended to CAZymes and assist in substrate binding and stimulate the catalytic efficiency of the enzymes [21]. *Paenibacillus* species can produce some distinct variations in the numbers and families of CAZymes. These variations also explain the good adaptability of *Paenibacillus* species to different circumstances [22].

Thirty-eight GHs were distributed to characterize starch, chitin, and cellulose degradation based on functional categorization (Table 3). Starch degrading activity was also observed in the experiment (Fig. 3B, the chitin and cellulose degradation activity tests were not carried out). Amongst the starch degrading enzymes, 13 α-amylase from the GH13 family and 2 β-amylase from the GH14 family were represented. The phylogenetic tree of these deduced amylase showed that there were differences (Fig. 3C) and only five amylase amino acid sequences had more than 70% similarity with sequences available in the GenBank database (Table S2).

**Table 3:** Starch, cellulose and chitin degrading enzymes predicted in the genome of the strain YPG26^T^ and their CAZymes family distribution

Substrate	Enzyme category	Enzyme name	EC number	Identified CAZymes families (number)
Starch	Amylase	α-amylase	3.2.1.1	GH13 (13)
	Amylase	β-amylase	3.2.1.2	GH14 (2)
	Amylase	γ-amylase	3.2.1.3	-
Chitin	Chitinase	Chitinase	3.2.1.14	GH18 (3), GH19 (3), GH23 (3)
Cellulose	Cellulase	α-glucosidase	3.2.1.20	GH31 (1), GH4 (1)
	Cellulase	β-glucosidase	3.2.1.21	GH1 (4), GH5 (2)
	Cellulase	Cellobiohydrolase	3.2.1.91	GH6 (1)
	Cellulase	Endoglucanase	3.2.1.4	GH5 (2), GH6 (1)

### Antimicrobial activity

**Fig. 4:**
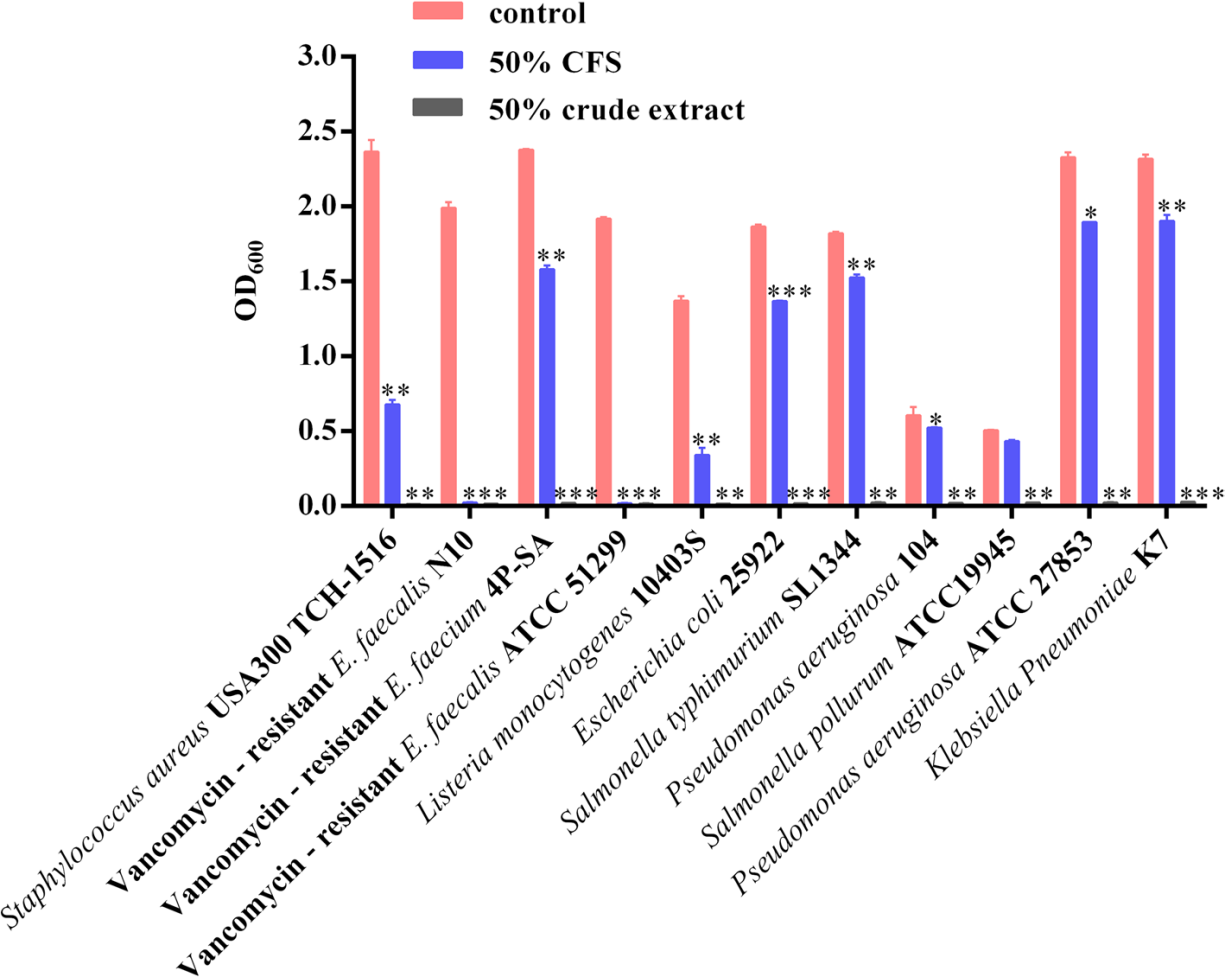
Antimicrobial activity of the strain YPG26^T^ against different pathogens. (Antimicrobial activity of the strain YPG26^T^ against pathogens after 6 h incubation with or without CFS and crude extract of the strain YPG26^T^, were determined by optical density (600 nm) measurements. Two biological replicates were set and values are expressed as mean ± SEM. Data were analyzed by student's t-test, *p<0.05, **p<0.01, ***p<0.001)

There is a large variety of antimicrobial substances produced by *Paenibacillus* species, which can target a range of human pathogens and plant pathogenic fungi [23]. Similarly, an in vitro antibacterial activity assay demonstrated some *Enterococcus* species are inhibited by cell-free supernatant (CFS) of *P. jilinensis* YPG26^T^, but other pathogens were not inhibited by the agar diffusion method (Table 4). We suspected that the concentration of antimicrobial substances in CFS was too low to measure. Thus, we prepared crude extract by saturated ammonium sulfate precipitation, and the antimicrobial activity was detected by growth determinations after co-culture with CFS and crude extract. The results showed that the growth of the majority of tested pathogenic bacteria was inhibited significantly after co-culturing with CFS compared to control cultures incubated without CFS, and the crude extract showed strong growth inhibition (Fig. 4), which indicated *P. jilinensis* YPG26^T^ can produce broad-spectrum antimicrobial substances.

**Table 4:** Antimicrobial activity test of the strain YPG26^T^ cell-free supernatant

Indicator strain	zone of inhibition diameter(mm)
*Enterobacter saccharolyticus* B2	13.45 ± 0.17
*Enterococcus faecium* M8-3	13.15 ± 0.43
*Enterococcus faecalis* M8-5	11.97 ± 0.23
Vancomycin—resistant *E. faecium* 4P-SA	11.41 ± 0.14
Vancomycin—resistant *E. faecalis* N10	10.10 ± 0.07
*Enterococcus hirae* M8-1	10.38 ± 0.03
*Staphylococcus aureus* USA300 TCH-1516	-
*Listeria monocytogenes* 10403S	-
*Escherichia coli* O78	-
*Salmonella typhimurium* SL1344	-
*Salmonella pollurum* ATCC19945	-
*Klebsiella Pneumoniae* K7	-

### Genome mining for BGCs of antimicrobial compounds

**Fig. 5:**
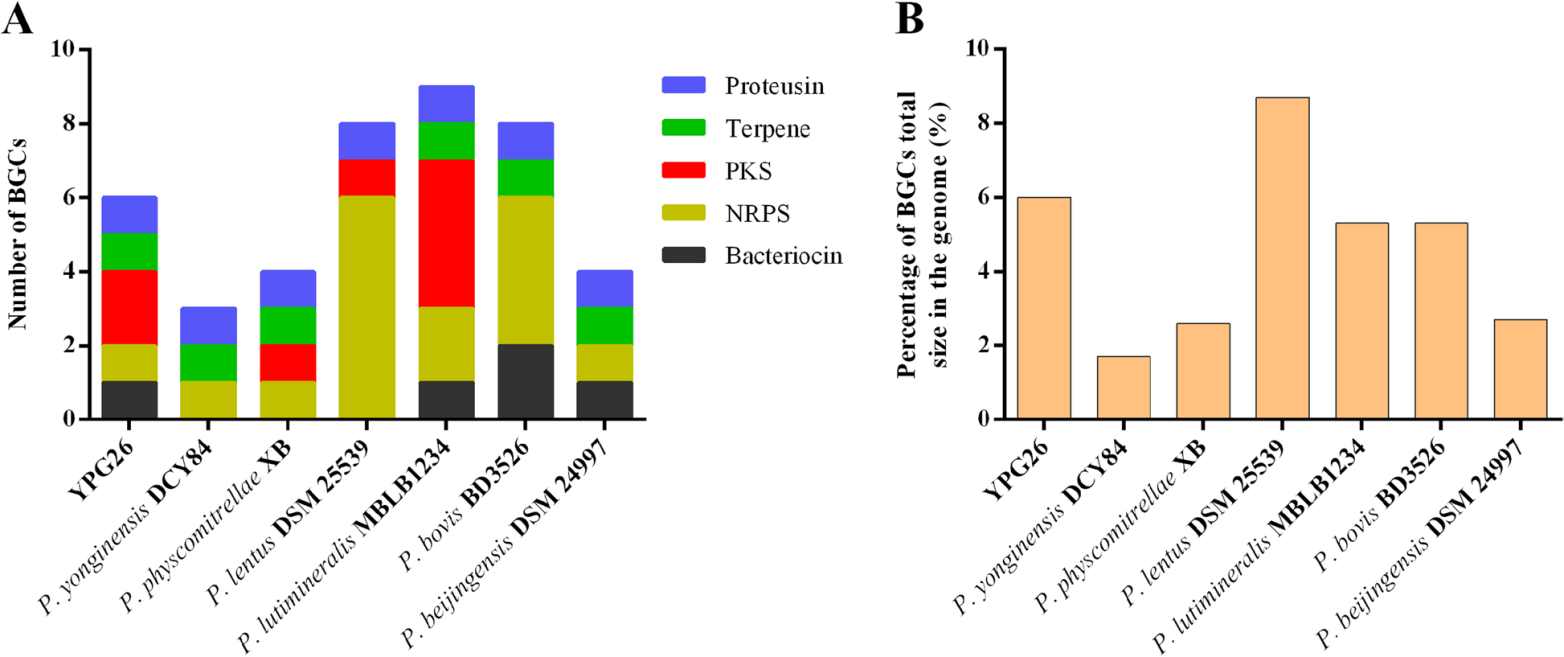
BGCs harbored by the different *Paenibacillus* species (**A**) Number of identified BGCs; (**B**) Percentage of BGCs total size in the whole genome

Genomic screening for biosynthetic gene clusters (BGCs) of antibacterial substances has been increasingly utilized in natural product discovery due to the large amount of bacteria whole-genome sequencing data available [24]. The genome mining indicated the presence of BGCs coding for 6 antimicrobial substances in the genome of *P. jilinensis* YPG26^T^: NRPS, PKS, lanthipeptide, proteusin, siderophore, and terpene (Fig. 5A), which all have low similarity with the known BGCs. The total size of all BGCs was approximately 238 kb, which accounted for 6.0% of the strain YPG26^T^ genome (Fig. 5B). Similarly, a previous study found the great number of varieties of BGCs in *Paenibacillus* and *Bacillus* strains by genome mining [25].

**Fig. 6:**
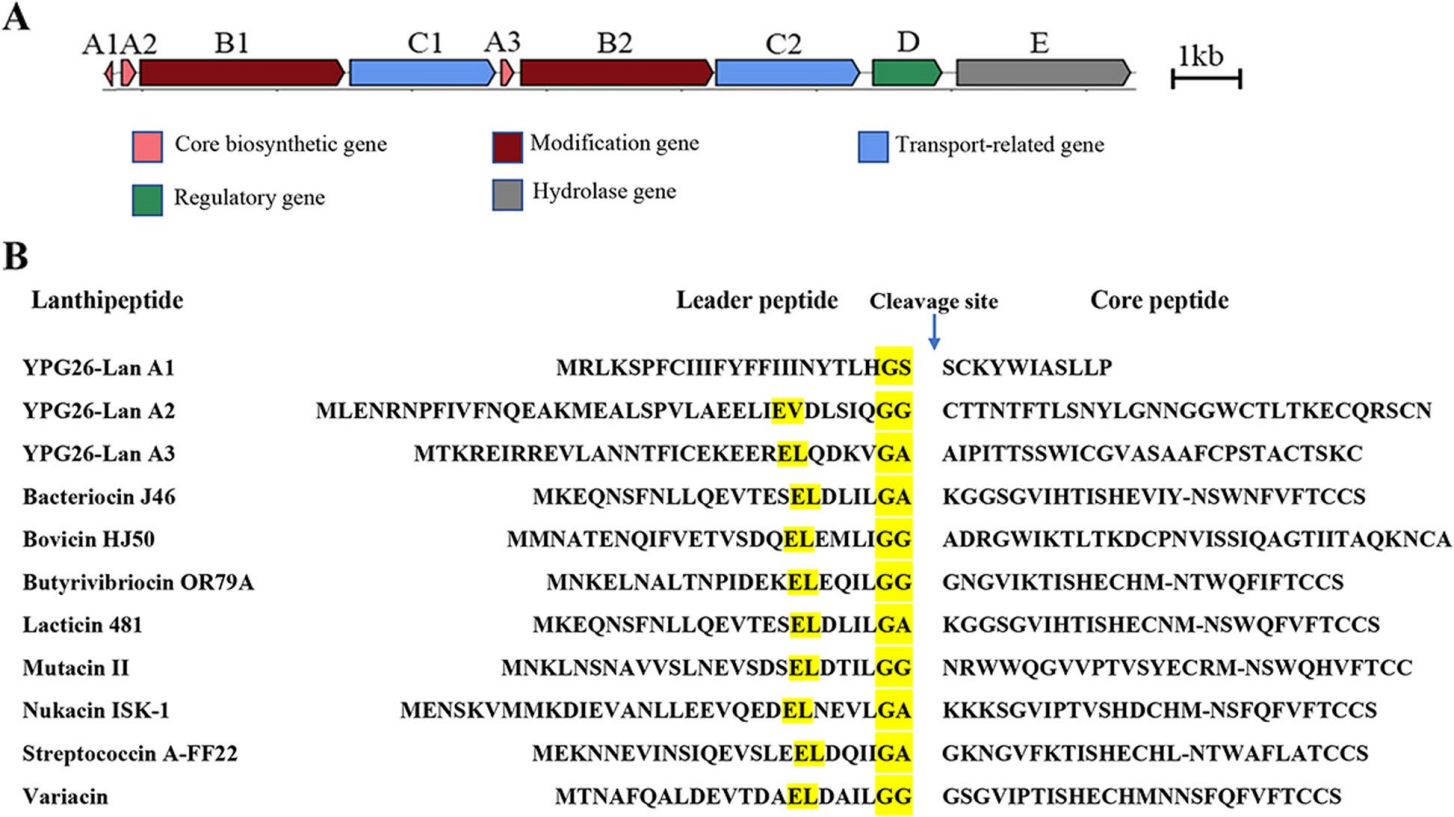
Putative BGCs of antimicrobial substances of the strain YPG26^T^ (**A**) Putative lanthipeptide BGCs of the strain YPG26^T^; **B** Multiple sequence alignment of the precursor peptide of the strain YPG26^T^ lanthipeptide with the precursor peptide of known class II lanthipeptide, conserved residues are highlighted in yellow

Experiments with proteinase processing revealed the proteinaceous nature of the antimicrobial compounds of *P. jilinensis* YPG26^T^, indicating that they could be bacteriocin-like substances, which was most likely to match the class II lanthipeptide BGCs with 3 core biosynthetic genes (named YPG26-lan A1, A2, and A3, respectively, Table S3) in the genome of *P. jilinensis* YPG26^T^ (Fig. 6A). The amino acid sequences of precursor peptides encoded by the three core biosynthetic genes were compared with other known class II lanthipeptides: bacteriocin J46, bovicin HJ50, butyrivibriocin OR79A, lacticin 481, mutacin II, nukacin ISK-1, steptococcin A-FF22, and variacin. The results showed that YPG26-lan A2 and YPG26-lan A3 have similar structural features to known lanthipeptides (Fig. 6B). The antibacterial activity of *P. jilinenesis* YPG26^T^ was likely conferred by YPG26-lan A2 or A3, or both. Subsequently, we will conduct further experiments to confirm the putative core biosynthetic genes of the lanthipeptide.

### Antibiotic-resistant genes analysis

An unreasonable use of antibiotics may change the intestinal flora, as a result, some commensal strains acquire antibiotic resistant genes (ARGs) in order to survive in the intestinal tract, which may increase public health risk in the future. Therefore, evaluation and monitoring of antibiotic resistant genes is an important measure to prevent resistance transfer [26]. Due to some bacteria’s slow growth or hard-to-culture, the use of whole-genome sequencing for antibiotic susceptibility testing has gradually become a powerful alternative [27]. The results revealed that 10 putative antibiotic resistance genes in the genome of *P. jilinensis* YPG26^T^ were responsible for resistance to antibiotics, including 8 efflux pump genes, 1 LlmA 23S ribosomal RNA methyltransferase gene, and 1 nucleoside resistance protein (tmrB) gene (Table S4). Culture-based antimicrobial susceptibility testing is still the primary method employed by clinical laboratories [27]. Thus, the sensitivity of *P. jilinensis* YPG26^T^ to different antibiotics has also been verified experimentally using the commercial antibiotics-discs diffusion method. It is a wonder that, the results showed sensitivity to all antibiotics tested (Table 5). This indicated that the strain YPG26^T^ has good safety.

**Table 5:** Antimicrobial susceptibility test of the strain YPG26^T^

Antibiotics	Disc conc(ug)	Diameter (mm)	Sensitivity/resistance
Penicillin	10U	38.35	S
Ampicillin	10	22.93	S
Cefuroxime	30	49.29	S
Ceftriaxone	30	42.37	S
Cefoperazone	75	46.08	S
Ceftazidime	30	31.80	S
Clindamycin	2	29.55	S
Chloramphenicol	30	33.73	S
Amikacin	30	33.96	S
Gentamicin	10	33.45	S
Kanamycin	30	30.16	S
Tetracycline	30	36.63	S
Erythromycin	15	40.06	S
Norfloxacin	10	34.44	S
Ofloxacin	5	38.61	S
Ciprofloxacin	5	39.73	S
Vancomycin	30	26.34	S

## Conclusion

In this study, we identified a novel *Paenibacillus* species isolated from the chicken intestine. The assembled genome analysis revealed that large numbers of carbohydrate degrading enzymes (CAZymes) genes and biosynthetic gene clusters (BGCs) of antimicrobial compounds are encoded in the genome. These genomic characteristics provid a better opportunity for understanding the intestinal niche adaption and biosynthetic potential of *Paenibacillus*, which will be indispensable for the direction of application in pharmaceutics, agriculture, or industry. Meanwhile, this study also contributed to the understanding of the genome features of intestinal uncultured bacteria.

## Methods

### Collection of microorganisms and isolation

Chicken fecal microorganisms (Changchun, Jilin Province, China) were serially diluted in sterile phosphate-buffered saline (PBS) solution and spread on yeast proteose glucose (YPG) agar plates with modifications (g/per 1000 ml: Tryptone 20.0, Yeast extract 10.0, Glucose 5.0, NaCl 0.08, L-cysteine 0.5, CaCl_2_ 0.008, MgSO_4_ 0.008, NaHCO_3_ 0.4, starch 5.0, pectin 0.5, Agar 15.) [28] and cultivated in anaerobic conditions (85% N_2_, 10% H_2_, 5% CO_2_) for 3 days at 37℃, the strain YPG26 was purified by subculturing. After isolation and purification, unless otherwise indicated, YPG26 was routinely cultured aerobically on tryptic soy broth (TSB, Qingdao Hope Biotechnology Co., Ltd, China). The strain YPG26^T^ was deposited in the China Center for Type Culture Collection (CCTCC) under the accession number CCTCC M2020899^T^.

### Phylogenetic analysis

The 16S rRNA gene of the strain YPG26^T^ was amplified and sequenced as previously described [29, 30]. Briefly, bacterial cells were dissolved in PCR lysis buffer (Takara, Japan). The genomic DNA was extracted using a commercial genomic DNA extraction kit, and the 16S rRNA gene was amplified using the universal bacterial primers 27F and 1492R [31]. The PCR product was sequenced, and the 16S rRNA gene sequence was compared with sequences available in GenBank by the nucleotide BLAST to determine an approximate phylogenetic affiliation. *Bacillus subtilis* DSM 10 (GenBank accession no. AJ276351) was used as an outgroup. The phylogenetic tree was set up using the neighbor-joining method and the maximum likelihood method in the MEGA 11.0 software [32], and the topologies were evaluated using the bootstrap resampling method with 1000 replications.

### Phenotypic characterization

Cell growth of the strain YPG26^T^ was monitored by measuring the optical density at 600 nm as previously described [33, 34], NaCl tolerance was measured in TSB medium supplemented with NaCl (i.e., the concentration of NaCl was 0.5–5%, w/v), and growth at different pH (2.0–10.0) and temperatures (4–50℃) was also tested. Cell morphology and the flagellum type were observed by transmission electron microscopy. The gram reaction was determined using the gram stain kit (Hangzhou Microbial Reagent Co., Ltd, China). Catalase activity was measured by the generation of bubbles in a 3% (v/v) H_2_O_2_ solution. Carbon source tests and biochemical tests were performed using the bacterial biochemical identification tube (Hangzhou Microbial Reagent Co., Ltd, China). Growth under anaerobic circumstances was measured in the anaerobic incubator (AL-B, LABIOPHY, Dalian, China) in an atmosphere of 85% N_2_, 10% CO_2_, and 5% H_2_ at 37 °C.

### Whole-genome sequencing, assembly, and annotation

Total genomic DNA of the strain YPG26^T^ was extracted using the bacteria DNA kit (TianGen Biotech (Beijing) Co. Ltd, China). The quality and quantity of genomic DNA were evaluated using agarose (Invitrogen, USA) gel electrophoresis and a Qubit fluorometer (Thermofisher, USA), respectively. Use the DNA library prep kit (NEB, USA) for sequencing library preparation. The whole genome of the strain YPG26^T^ was performed using the PE150 PacBio Sequel platform and Illumina NovaSeq at the Beijing Novogene Bioinformatics Technology Co., Ltd. Assembly was completed by using SMRT link v5.0.1. Furthermore, the corrected assembly result was filtered with the base minimum mass value of 20. Finally, based on the overlap between the head and the tail, confirmed whether the genome sequence formed a circle or not and corrected the initial site by blasing with the DNA database. The annotation of the assembled genome was performed using rapid annotation subsystems technology (RAST) [35]. The tRNA and rRNA were predicted using the tRNAscan-SE [36] and rRNAmmer [37], respectively. For functional gene annotation, GO (gene ontology) [38] and COG (clusters of orthologous groups) database [39] were used.

### Genome-genome distance, and average nucleotide identity

The digital DNA-DNA hybridization (dDDH) values among the strain YPG26^T^ and other members of the *Paenibacilllus* were calculated using the genome-to-genome distance calculator 3.0 (GGDC) [40]. Furthermore, the pairwise genome similarity was assessed using the average nucleotide identity (ANI) calculated with the JSpeciesWS web (http://jspecies.ribohost.com/jspeciesws/#analyse) [41].

### CAZymes identification and mining of starch degrading genes

The carbohydrate-active enzymes (CAZymes) of the strain YPG26^T^ were identified and classified using the carbohydrate-active enzymes database [42]. The starch-degrading genes were further revealed by cross-checking with the annotations available in the database. In vitro determination of amylase activity was carried out according to previously described [43]. Briefly, starch agar media (g/per 1000 ml: Tryptone 10.0, Yeast extract 10.0, KH_2_PO_4_ 5.0, soluble starch 3.0, Agar 15) was used, 10 μL with 10^8^ cfu/mL of the strain YPG26^T^ was placed on the center of the plate and incubated at 37 °C for 48 h. For visualization of the zone of clearance, the plate was flooded with 2 mL of Gram’s iodine solution.

### Antimicrobial activity

To prepare cell-free supernatants (CFS), the strain YPG26^T^ was cultured in TSB at 37℃ for 10 h with shaking at 200 rpm. After incubation, bacterial suspension was centrifuged at 8000 g for 10 min. Supernatants were collected and filter-sterilized with a 0.22 μm filter (Millipore, USA). The antimicrobial activity of CFS was evaluated initially according to the effect of CFS on the viability of the pathogenic bacteria [44] and appropriate modifications. Briefly, overnight pathogenic cultures were sub-cultured in TSB at 37℃ to logarithmic phase. Adjusting OD_600_ to 0.02, 50 µL of each pathogenic bacteria was added per well in a 96-well plate, followed by 50 µL CFS of the strain YPG26^T^ or TSB medium alone (negative control). The cultures were incubated at 37℃ for 6 h, the absorbance values at 600 nm were measured using a microplate reader (Eppendorf, Germany). Relative to the absorbance value of the negative control, the absorbance value of adding CFS reflects the antimicrobial activity of the strain YPG26^T^. Two biological replicates were set.

To prepare crude extracts, the saturated ammonium sulfate was slowly added to the supernatant to reach 70% saturation. The precipitate was collected after standing at 4℃ overnight and 10,000 g was centrifugated for 20 min. It was then redissolved in sterile distilled water, and dialyzed extensively with sterile distilled water to remove ammonium sulfate. Finally, the crude extract was freeze-dried, then redissolved in sterile distilled water and the antibacterial activity was detected by the same method as the CFS antibacterial activity evaluation.

### Genome mining for BGCs of antimicrobial compounds

Genome mining for biosynthetic gene clusters (BGCs) of antimicrobial compounds was carried out using the antiSMASH version 6.0.1 [45]. BGCs that differed from previously reported ones by less than 70% were considered novel [46]. The putative core biosynthetic genes of bacteriocin were further confirmed with NCBI protein BLAST. Multiple alignments of amino acid sequences of the deduced precursor peptide with other known lanthipeptides were performed using MEGA 7.0 software.

### Antibiotic-resistant genes analysis

Antibiotic-resistant genes of the strain YPG26^T^ were identified by comparing whole-genome sequences against the comprehensive antibiotic research database (CARD) [47]. The antibiotic susceptibility of the strain YPG26^T^ was determined by modifying the disc diffusion test as previously described [48]. Briefly, commercial antibiotic discs (Hangzhou Microbial Reagent Co., Ltd, China) were placed on MH agar (Qingdao Hope Biotechnology Co., Ltd, China) plates inoculated with strain YPG26^T^ (10^8^ CFU). The plates were incubated at 37℃ for 24 h. Inhibition zone diameters were measured and referred to the Clinical and Laboratory Standards Institute (CLSI) interpretative zone diameters for disc diffusion. The results were described in terms of resistance (R), moderate resistance (MR), or susceptibility (S).

## Supplementary Information

**Additional file 1.** 

## Data Availability

The 16S rRNA sequence is available in GenBank with the accession OK324374 (https://www.ncbi.nlm.nih.gov/nuccore/OK324374). And the whole genome data are available at DDBJ/EMBL/GenBank under the bioproject accession PRJNA766864 (https://www.ncbi.nlm.nih.gov/bioproject/?term=PRJNA766864).
