# Supplementary material for: Genome mining reveals polysaccharide-degrading potential and new antimicrobial gene clusters of novel intestinal bacterium Paenibacillus jilinensis sp. nov
Source: BMC Genomics. 2022 May 19;23:380. doi: 10.1186/s12864-022-08623-4 (PMC9118873; doi:10.1186/s12864-022-08623-4)
Supplement: Supplementary file 1 — Additional file 1. [file 12864_2022_8623_MOESM1_ESM.docx]

**Supplementary Information**

**Genome mining reveals polysaccharide-degrading potential and new antimicrobial gene clusters of novel intestinal bacterium *Paenibacillus jilinensis* sp. nov.**

**Ke Ma^1†^, Wei Chen^1†^, Shi-Qing Yan^1^, Xiao-Qi Lin^1^, Zhen-Zhen Liu^1^, Jia-Bao Zhang^1^, Yu Gao^1^, Yong-Jun Yang^1^***

^1^Key Laboratory of Zoonosis Research, Ministry of Education, College of Veterinary Medicine, Jilin University, Changchun, China.

***Correspondence:** Yong-Jun Yang, youngjune@jlu.edu.cn; No. 5333 Xi'an Road, Changchun 130062, China. Tel: 86-431-87836424.

†These authors have contributed equally to this work.

**Table and Figure Legends**

**Table S1.** Average nucleotide identity (ANI) and digital DNA–DNA hybridization (dDDH) between strain YPG26^T^ and other species of *Paenibacillus*.

**Table S2.** The deduced amylase from the genome of the strain YPG26^T^.

**Table S3.** The putative BGCs genes of antimicrobial substances of the strain YPG26^T^.

**Table S4.** The predicted antibiotic resistance genes in the genome of the strain YPG26^T^.

**Figure S1.** Phenotypic characteristics of the strain YPG26^T^.

**Figure S2.** Functional annotation of the strain YPG26^T^ genome. (A) COG functional annotation; (B) KEGG functional annotation; (C) GO functional annotation.

**Supplementary Table 1.** **Average nucleotide identity (ANI) and digital DNA–DNA hybridization (dDDH) between strain YPG26^T^ and other species of *Paenibacillus***.

The whole genome sequences of other *Paenibacillus* species were derived from NCBI GenBank database (https://www.ncbi.nlm.nih.gov/genome/?term=paenibacillus).

| Strain | RefSeq assembly Accession no. | YPG26 | |
| --- | --- | --- | --- |
|  |  | ANI（%） | dDDH（%） |
| *Paenibacillus yonginensis* strain DCY84 | NZ_CP014167.1 | 71.07 | 14 |
| *Paenibacillus physcomitrellae* strain XB | NZ_CP022584.1 | 71.02 | 13.8 |
| *Paenibacillus lentus* strain DSM 25539 | NZ_CP034248.1 | 70.58 | 13.9 |
| *Paenibacillus lutimineralis* strain MBLB1234 | NZ_CP034346.1 | 70.52 | 13.5 |
| *Paenibacillus sabinae* T27 | NZ_CP004078.1 | 70.41 | 13.5 |
| *Paenibacillus cellulositrophicus* strain KACC 16577 | NZ_CP045295.1 | 70.29 | 13.6 |
| *Paenibacillus ihbetae* strain IHBB 9852 | NZ_CP016809.1 | 70.21 | 13.5 |
| *Paenibacillus xylanilyticus* strain W4 | NZ_CP018620.1 | 70.02 | 13.3 |
| *Paenibacillus lautus* strain E7593-69 | NZ_CP032412.1 | 70 | 13.4 |
| *Paenibacillus donghaensis* strain KCTC 13049 | NZ_CP021780.1 | 69.95 | 13.4 |
| *Paenibacillus jilunlii* strain KACC 16679 | NZ_CP048429.1 | 69.94 | 13.4 |
| *Paenibacillus stellifer* strain DSM 14472 | NZ_CP009286.1 | 69.9 | 13.4 |
| *Paenibacillus durus* strain DSM 1735 | NZ_CP009288.1 | 69.89 | 13.4 |
| *Paenibacillus graminis* strain DSM 15220 | NZ_CP009287.1 | 69.89 | 13.4 |
| *Paenibacillus borealis* strain DSM 13188 | NZ_CP009285.1 | 69.88 | 13.3 |
| *Paenibacillus riograndensis* SBR5 isolate SBR5(T) | NZ_LN831776.1 | 69.87 | 13.4 |
| *Paenibacillus glucanolyticus* strain DSM 5162 | NZ_CP015286.1 | 69.86 | 13.6 |
| *Paenibacillus kribbensis* strain AM49 | NZ_CP020028.1 | 69.73 | 13.4 |
| *Paenibacillus brasilensis* strain KACC 13842 | NZ_CP045298.1 | 69.7 | 13.5 |
| *Paenibacillus xylanexedens* strain PAMC 22703 | NZ_CP018620.1 | 69.7 | 13.3 |
| *Paenibacillus terrae* HPL-003 | NC_016641.1 | 69.6 | 13.4 |
| *Paenibacillus bovis* strain BD3526 | NZ_CP013023.1 | 69.55 | 13.4 |
| *Paenibacillus polymyxa* E681 | NC_014483.2 | 69.54 | 13.5 |
| *Paenibacillus peoriae* strain HS311 | NZ_CP011512.1 | 69.51 | 13.4 |
| *Paenibacillus uliginis* N3/975 | LT840184.1 | 69.47 | 13.4 |
| *Paenibacillus swuensis* strain DY6 | NZ_CP011388.1 | 69.41 | 13.4 |
| *Paenibacillus odorifer* strain DSM 15391 | NZ_CP009428.1 | 69.18 | 13.3 |
| *Paenibacillus beijingensis* strain DSM 24997 | NZ_CP011058.1 | 68.74 | 13.2 |
| *Paenibacillus guangzhouensis* strain KCTC 33171 | NZ_CP045293.1 | 68.66 | 13.3 |
| *Paenibacillus thiaminolyticus* strain NRRL B-4156 | NZ_CP041405.1 | 68.66 | 13.2 |
| *Paenibacillus protaetiae* strain FW100M-2 | NZ_CP035492.1 | 68.56 | 13.3 |
| *Paenibacillus albus* strain 18JY67-1 | NZ_CP034437.1 | 68.55 | 13.2 |
| *Paenibacillus crassostreae* strain LPB0068 | NZ_CP017770.1 | 68.53 | 13.4 |
| *Paenibacillus antarcticus* strain KACC 11469 | NZ_CP043611.1 | 68.48 | 13.4 |
| *Paenibacillus mucilaginosus* KNP414 | NC_015690.1 | 68.46 | 13.2 |
| *Paenibacillus chitinolyticus* strain KCCM 41400 | NZ_CP026520.1 | 68.34 | 13.2 |
| *Paenibacillus naphthalenovorans* strain 32O-Y | NZ_CP013652.1 | 68.18 | 13.2 |
| *Paenibacillus baekrokdamisoli* strain KCTC 33723 | NZ_AP019308.1 | 68 | 13.1 |
| *Paenibacillus psychroresistens* strain ML311-T8 | NZ_CP034235.1 | 67.84 | 13.2 |
| *Paenibacillus larvae subsp. larvae* strain ATCC 9545 | NZ_CP019687.1 | 67.56 | 13.2 |

**Supplementary Table 2.** **The deduced amylase from the genome of the strain YPG26^T^.**

Sequence alignment of the deduced amylase sequence of the strain YPG26^T^ were performed with sequences available in the GenBank database.

|  | YPG26_  Gene_id | Identity (%) | GenBank_  Accession | CAZymes_  family |
| --- | --- | --- | --- | --- |
| α-Amylase | GM000512 | 56.6 | APO47284.1 | GH13_14 |
|  | GM000514 | 74.9 | AWB43174.1 | GH13_20 |
|  | GM000649 | 75.7 | AIQ34118.1 | GH13_31 |
|  | GM000819 | 41.9 | AWK41767.1 | GH13_3 |
|  | GM000825 | 69.5 | AIQ34761.1 | GH13_12 |
|  | GM000893 | 59.6 | AIQ36212.1 | GH13_36 |
|  | GM001245 | 65 | AIQ65105.1 | GH13_20 |
|  | GM002325 | 65.5 | ANY75663.1 | GH13_14 |
|  | GM002347 | 79.6 | AWB44458.1 | GH13_31 |
|  | GM002354 | 80.3 | AWV36638.1 | GH13_31 |
|  | GM002792 | 60.6 | AFH61936.1 | GH13 |
|  | GM003448 | 57.1 | AHO16406.1 | GH13_11 |
|  | GM003615 | 79.1 | AIQ61435.1 | GH13_5 |
| β-Amylase | GM003086 | 69.3 | AFH60845.1 | GH14 |
|  | GM003767 | 41.4 | AAA23204.1 | GH14 |

**Supplementary Table 3.** **The putative BGCs genes of antimicrobial substances of the strain YPG26^T^.**

The putative BGCs gene’s function were identified by comparing gene sequences available in the GenBank database.

| Gene | YPG26_  Gene_ Location: | Gene function | GenBank_  Accession | Identity |
| --- | --- | --- | --- | --- |
| A1 | 463,481 - 463,591 | / | / | / |
| A2 | 463,709 - 463,915 | plantaricin C family lantibiotic | WP_199794786.1 | 83.82% |
| A3 | 469,340 - 469,519 | class II lanthipeptide (LchA2/BrtA2 family) | WP_146113448.1 | 75.00% |
| B1 | 463,983 - 467,006 | type 2 lantipeptide synthetase LanM | WP_158702647.1 | 75.50% |
| C1 | 467,097 - 469,247 | ATP-binding cassette domain-containing protein | WP_158702646.1 | 77.06% |
| B2 | 469,633 - 472,479 | type 2 lantipeptide synthetase LanM | WP_106765798.1 | 67.41% |
| C2 | 472,523 - 474,652 | ABC transporter | WP_106765797.1 | 80.93% |
| D | 474,854 - 475,870 | LacI family DNA-binding transcriptional regulator | WP_127199122.1 | 83.09% |
| E | 476,097 - 478,667 | glycoside hydrolase family 2 protein | WP_127199121.1 | 86.44% |

**Supplementary Table 4.** **The predicted antibiotic resistance genes in the genome of the strain YPG26^T^.**

Antibiotic-resistant genes were identified by comparing whole-genome sequences against the comprehensive antibiotic research database (CARD).

| YPG26_  Gene_id | Gene name | ARO | Identity |
| --- | --- | --- | --- |
| GM000931 | salA | 3003749 | 0.30 |
| GM002278 | lrfA | 3003730 | 0.33 |
| GM003525 | efrA | 3003948 | 0.46 |
| GM003758 | mepA | 3000026 | 0.47 |
| GM003216 | tmrB | 3003059 | 0.52 |
| GM002058 | blt | 3003551 | 0.53 |
| GM002429 | sav1866 | 3000489 | 0.55 |
| GM002080 | lsaC | 3003112 | 0.60 |
| GM002716 | TaeA | 3003986 | 0.72 |
| GM001870 | LlmA 23S ribosomal RNA methyltransferase | 3003982 | 0.84 |

**
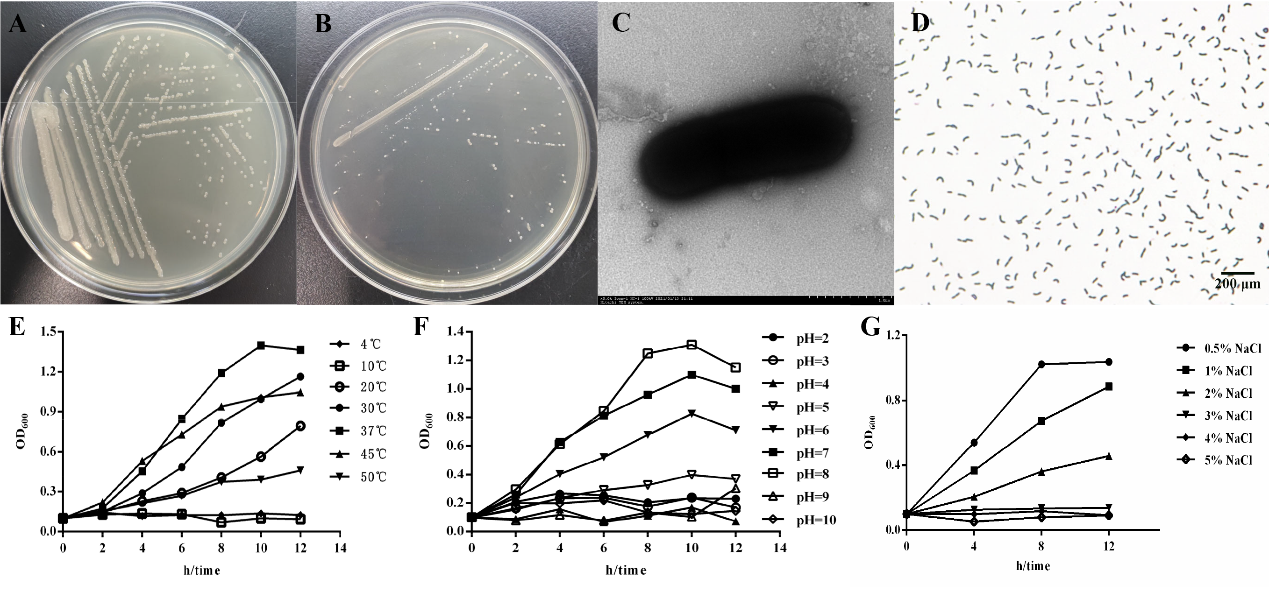
**

**Supplementary Figure 1** **Phenotypic characteristics of the strain YPG26^T^.**

**(A)** Colony morphology of the strain YPG26^T^ aerobic culture on TSB agar plate; **(B)** Colony morphology of the strain YPG26^T^ anaerobic culture on TSB agar plate; **(C)** Transmission electron microscopy (TEM) image of the strain YPG26^T^; **(D)** Gram staining of the strain YPG26^T^; **(E-G)** Growth curves of the strain YPG26^T^ at different temperature, pH, and NaCl concentration.


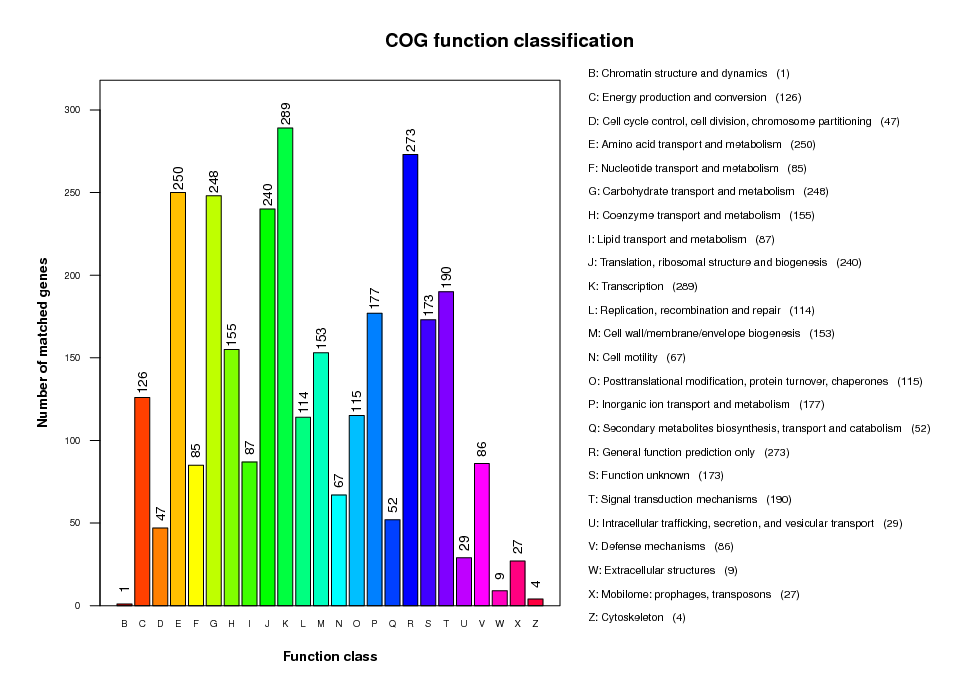


(A) COG functional annotation


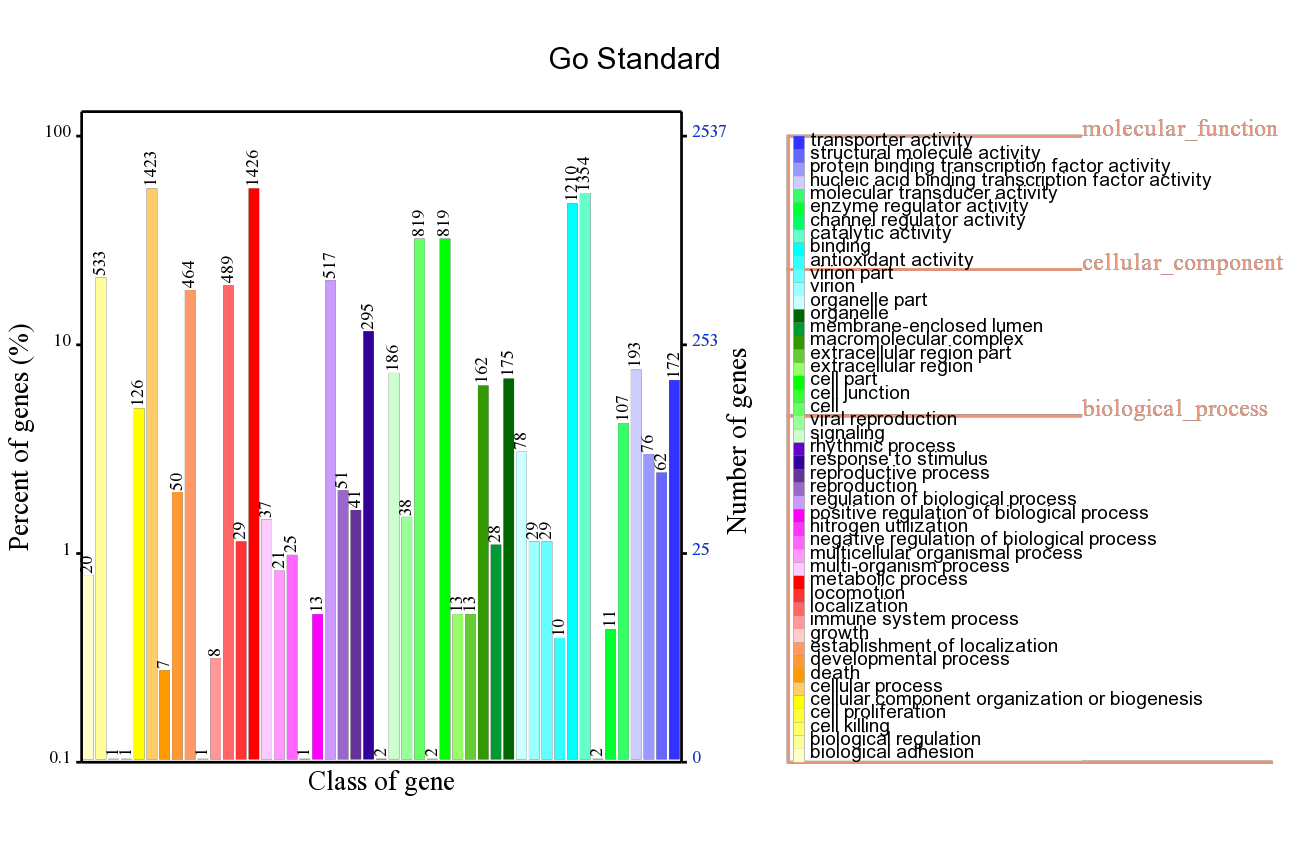


(B) GO functional annotation

**Supplementary Figure 2** **Functional annotation of the strain YPG26^T^ genome.**

**(A)** COG functional annotation; **(B)** GO functional annotation.
